# The outlook for science and health care in the UK: A bleak picture?

**DOI:** 10.1016/j.lanepe.2022.100560

**Published:** 2022-12-01

**Authors:** The Lancet Regional Health – Europe

It has been a turbulent few months for UK politics with the appointment of two different Prime Ministers since July 2022, and a flurry of resignations and dismissals of senior government ministers during the same period. Additionally, the UK economy is in a dire state, with inflation rising above 11% and a recession predicted that could be the longest since the 1920s. Amidst this economic crisis there is little hope that Rishi Sunak, the new Prime Minister of the UK, will be able to effectively prioritise science and health care related issues that have not been addressed since Brexit: insufficient scientific funding and a regressing National Health Service (NHS) that is grappling with 130,000 vacancies. Fixing the economy must of course be prioritised, but in doing so science and health care should not be underfunded or deprioritised.

After Brexit, the UK has been temporarily barred access to €95.5 billion of funding from the European Union (EU) Horizon programme, a key funding programme for research and innovation. Prior to Brexit, the UK was the second most successful country (after Germany) in receiving €7 billion of the Horizon 2020 research programme, which was more than the country's average contribution to the overall 2019 EU budget. The benefit of receiving substantial grant funding was reflected by the 5.4% increase in research articles published between 2016 and 2020, placing the UK third in global scientific research output, after the USA and China. However, without sufficient funding it is doubtful that the UK can continue to maintain this top position in future.

The absence of clarity and decisiveness from the UK Government on Plan B, the domestic alternative programme to Horizon Europe, has resulted in many researchers being faced with the ultimatum of either leaving the UK to work at an EU institution or losing their EU research grants. This will hamper the growth and innovation of science, making the UK an unattractive destination for budding researchers. The government has promised to protect the research and development budget with an increase to £20bn by 2024–25, however this may not be enough. As highlighted in a report published by the *British Medical Journal*, if the UK is to remain at the forefront of research and innovation, it is imperative to prioritise bilateral negotiations and deals with EU and other scientific institutions, implement policies to attract and retain research talent, invest in high quality health data infrastructure, and ensure that science and research is at the heart of its trade strategy.

Furthermore, since the exit of European Medicines Agency—the EU's drug regulator—from London to Amsterdam, the number of newly approved drugs for use in the UK has declined, indicating that the UK is becoming a less attractive place for industrial partners and drug manufacturers. Further evidence that industrial partners are reluctant to invest in the UK is demonstrated by the 41% reduction in industry-led clinical trials between 2017 and 2021. This declining trajectory represents a considerable loss of opportunity in the UK, both scientifically and economically, and should be of serious cause of concern to the present Government.

Regarding health care, at present there are 7 million people on the NHS waiting list for treatment. The COVID-19 pandemic was a contributing factor to the growing backlog, however, long waiting times pre-date the pandemic, and the inadequate funding and resources allocated by the previous and present Conservative governments have failed to meet the rising demand of the NHS. Chronic staff shortages and low staff retention are major problems with many health-care workers feeling forced to leave the NHS due to pay erosion and unbearable working conditions. A £3.3 billion increase in funding to NHS has been announced by the government last month, however, the NHS will still be in deficit of £7 billion in 2023–2024 due to inflation and will remain under financial strain. The NHS may have to cut back cancer care, GP provision, and mental healthcare if its budget is not increased to deal with inflation.

During Rishi Sunak's prime ministerial campaign, he promised to “secure the UK as a science and technology superpower”, but to honour this pledge, consistent and long-term commitment is required with a clear vision and roadmap. He has recognised the need for a science minister by appointing Nusrat Ghani to a post that was previously vacant for 3 months during Liz Truss' short-lived tenure. Although this post has been re-instated to a senior role, Ghani is not included within the cabinet of senior ministers who advise Sunak, perhaps indicating that science is not a high priority. It is crucial that the UK Government understands the urgency to restore the declining state of health care, science funding, and confidence of researchers in the UK because the UK's reputation as a leading global science and research powerhouse is under serious threat.

